# Antisense oligonucleotides against microRNA-21 reduced the proliferation and migration of human colon carcinoma cells

**DOI:** 10.1186/s12935-015-0228-7

**Published:** 2015-08-01

**Authors:** Yi-Jing Tao, Yong-ju Li, Wen Zheng, Juan-juan Zhao, Meng-meng Guo, Ya Zhou, Na-lin Qin, Jing Zheng, Lin Xu

**Affiliations:** Department of Immunology, Zunyi Medical College, Zunyi, 563003 Guizhou China; Department of Medical Physics, Zunyi Medical College, Zunyi, 563000 Guizhou China

**Keywords:** MicroRNA-21, Colon carcinoma, Antisense oligonucleotides (ASO), Phosphatase and tensin homolog (PTEN)

## Abstract

**Background:**

Colon carcinoma is one of the commonly tumors that threaten human beings as its highly morbidity and mortality. Recent evidences suggested that microRNA-21 (miR-21) played an important role in the development of colon carcinoma and might be a potential biological marker for the diagnosis and prognosis of colon carcinoma. However, the potential effect of miR-21 based therapeutic studies in colon carcinoma remains to be fully elucidated.

**Methods:**

In present study, we constructed an eukaryotic expression vector encoding antisense oligonucleotides against miR-21 (termed as p-miR-21-ASO) and the expression of miRNA-21 in human colon cancer was detected by Real-time PCR. To assess its possible effect on the proliferation and migration capacity of human colon carcinoma cells in vitro, CCK-8 assay, colony formation assay and cell invasion, as well as migration assay, were performed respectively. Moreover, PTEN, one of target molecules of miRNA-21, was analyzed by Western blot and Fluorescence activated cell sorter assay. Finally, the transduction of AKT and ERK pathways in human colon carcinoma cells was determined by Western blot.

**Results:**

We found that transiently transfection of p-miR-21-ASO could efficiently decrease the relative expression of miR-21 in human colon carcinoma HCT116 cells, accompanied by impaired proliferation and clone formation. Furthermore, we found that down-regulation of miR-21 also could significantly abrogate the invasion and migration capacity in vitro, as well as the expression of vascular endothelial growth factor which is critical for the metastatic capacity of colon carcinoma cells. Mechanistic evidence showed that down-regulation of miR-21 increased the expression of its target molecule PTEN in HCT116 cells. Finally, we revealed that the expression level of both phosphor-ERK1/2 and phosphor-AKT also were altered.

**Conclusions:**

Therefore, our data suggested miR-21 ASO against miR-21 might be a useful strategy to alter the expression of miR-21 in colon carcinoma cells, which was helpful for the development of miR-21-based therapeutic strategies against clinical colon carcinoma.

**Electronic supplementary material:**

The online version of this article (doi:10.1186/s12935-015-0228-7) contains supplementary material, which is available to authorized users.

## Background

Colon carcinoma is one of the commonly tumors that threaten human beings as its highly morbidity and mortality [1, 2]. The development of colon carcinoma is a complex process that requires a series of integrated steps including cellular neoplastic transformation, unlimited growth, and the acquisition of invasive/metastatic properties, as well as immunologic escape [3, 4]. Although extensive investigation explored some important factors of colon carcinoma, the effect of various treatment approaches including surgical operation, chemotherapy and immune cell based therapy remains limited because of the complex process of development of colon carcinoma [5, 6]. Thus, new strategies are still required for achieving effective treatment of colon carcinoma, which might ultimately aid the clinical therapy for colon carcinoma patients.

MiRNA-21 is an important member of miRNAs, which located on chromosome 17q23-2 overlapping with the TMEM49 gene and is regulated through its promoter containing binding sites for AP-1 and PU.1 transcription factors [7]. Numbers of researches have been reported on miRNA-21 play a critical role in the development of kinds of tumors via a variety of molecular mechanisms [8, 9]. To colon carcinoma, recent evidences also suggested that miR-21 as an oncomiRNA molecule played an important regulator role in the development of colon carcinoma including the proliferation, invasion and metastatic potential of cancer cells. For instance, Drusco et al. reported that miRNA-21 might be a potential metastatic signature of colon cancer, and a useful marker distinguishing colon cancer recurrences to lymph nodes from liver, or colon cancer liver metastasis from primary hepatic tumor [10]. Similarly, Roy et al. found that overexpression of miR-21 could enhance the growth of colon cancer cells in vivo through down-regulation of PTEN [11]. Nangia-Makker et al. further reported that metformin combined with 5-fluorouracil and oxaliplatin in the treatment of colon carcinoma induced cell apoptosis in chemo-resistant HCT116 cells, which was associated with reduced expression of miRNA-21 [12]. In addition, Li et al. showed that miRNA-21 might be a useful biological marker which was closely related to the diagnosis and prognosis of colon carcinoma [7]. These researches indicated the important role of miR-21 in the development and the diagnosis, as well as prognosis of colon carcinoma. However, whether miR-21 may be used as a potential target in the biological therapy against colon carcinoma remains to be further elucidated.

To this aim, in present study, we constructed an eukaryotic expression vector encoding antisense oligonucleotides (ASOs) against miR-21 (termed as p-miR-21-ASO) and assessed its possible effect on the proliferation and migration capacity of human colon carcinoma cells and explored the related mechanism, which might be helpful for the development of miR-21-based therapeutic strategies against clinical colon carcinoma.

## Results

### MiRNA-21 ASO reduced the proliferation of human colon carcinoma cells

**Fig. 1:**
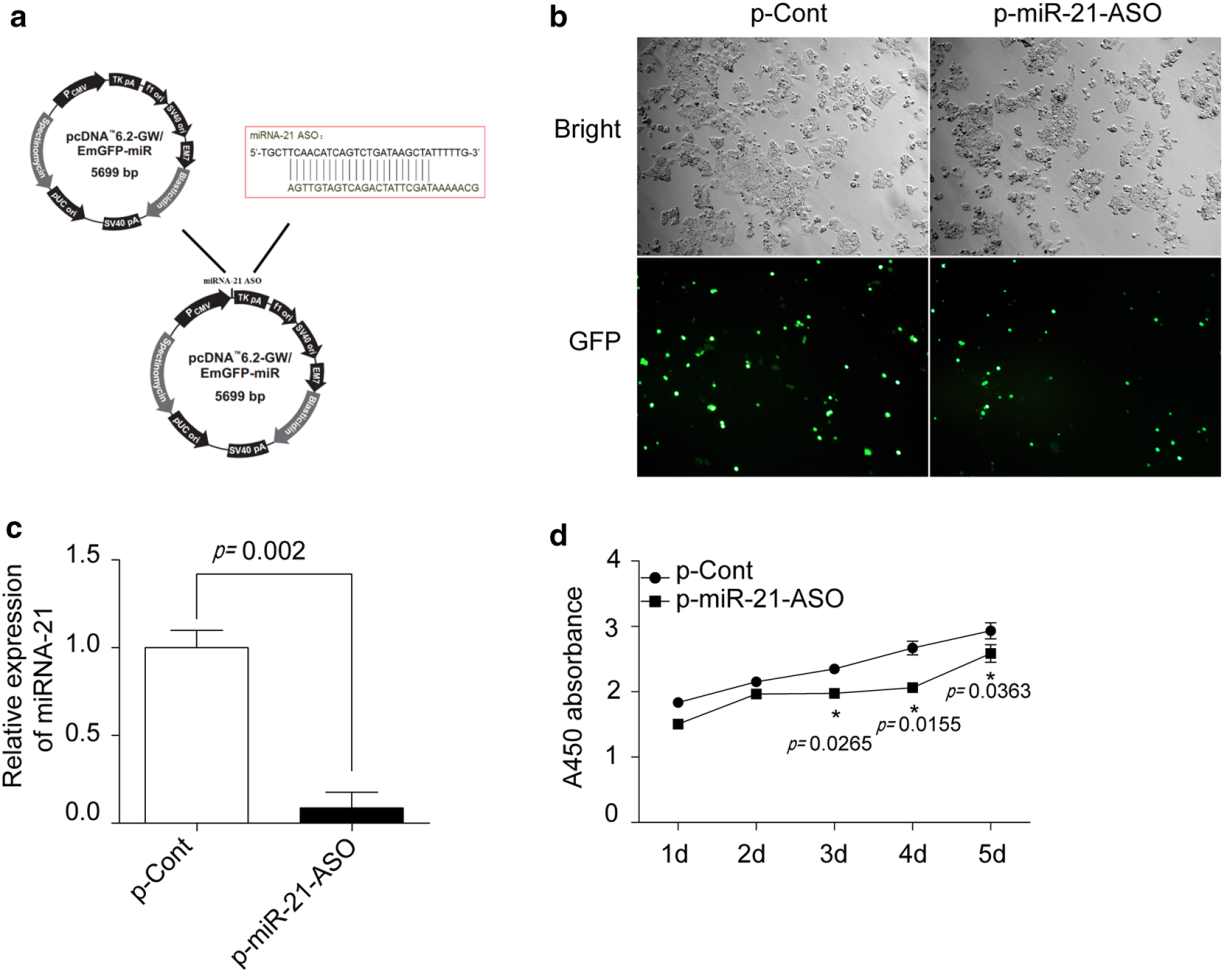
MiRNA-21 ASO reduced the proliferation of human colon carcinoma cells. **a** The schematic of a eukaryotic expression vector encoding antisense oligonucleotides against miR-21 (termed as p-miR-21-ASO). **b** Human colon carcinoma cell line HCT116 cells were cultured in 96 well plate and transiently transfected with p-miR-21-ASO or p-Cont (5 μg). After 48 h, the expression of GFP protein was observed by fluorescent microscopy. **c** The expression level of miR-21 in HCT116 cells was also determined by Real-time PCR assay and calculated. **d** At indicated time point, the proliferation of HCT116 cells in p-miR-21-ASO transfected group and p-Cont group also were detected by CCK-8 assay. One representative of three experiments was shown. *p < 0.05.

To investigate the possible effect of miR-21-based therapeutic strategy against human colon carcinoma cells, we firstly identified the relative expression of miRNA-21 in a set of three colon carcinoma cell lines and a normal colonic cell line using Real-time PCR assay. As shown in Additional file 1: Figure S1, miRNA-21 levels were up-regulated in all carcinoma cell lines compared with normal control (p < 0.05), which was consistent with previous report [7]. Then we constructed an eukaryotic expression vector encoding antisense oligonucleotides (ASOs) against miR-21 (termed as p-miR-21-ASO), and then transiently transfected into colon carcinoma cell line HCT116 cells (Fig. 1a). The transfection efficiency was verified by detecting the expression of GFP by fluorescence microscopy. As shown in Fig. 1b, the mean proportion of GFP positive cells was about 70% in each group at 48 h after transfection. As expected, real-time PCR analysis showed that the expression of miRNA-21 in p-miR-21-ASO transfection group was significantly decreased compared with that in p-Cont transfection group (Fig. 1c, p < 0.05), suggesting that miR-21 ASO could significantly reduce the expression of intrinsic miR-21 in colon carcinoma cells.

Importantly, we found that the proliferation of HCT116 was significantly decreased in p-miR-21-ASO transfected groups compared with that in control group (Fig. 1d, p < 0.05). To further confirm the effect of miR-21 ASO on the growth of colon carcinoma cells, we also detected the possible influence of miR-21 ASO on another colon carcinoma cell line SW620 cells which expressed high level of intrinsic miR-21 and obtained similar results (Additional file 2: Figure S2). Combining these data indicated that miR-21 ASO could effectively down-regulate the expression of miR-21 and successively reduce the proliferation capacity of colon carcinoma cells.

### MiRNA-21 ASO reduced the colony formation capacity of human colon carcinoma cells

**Fig. 2:**
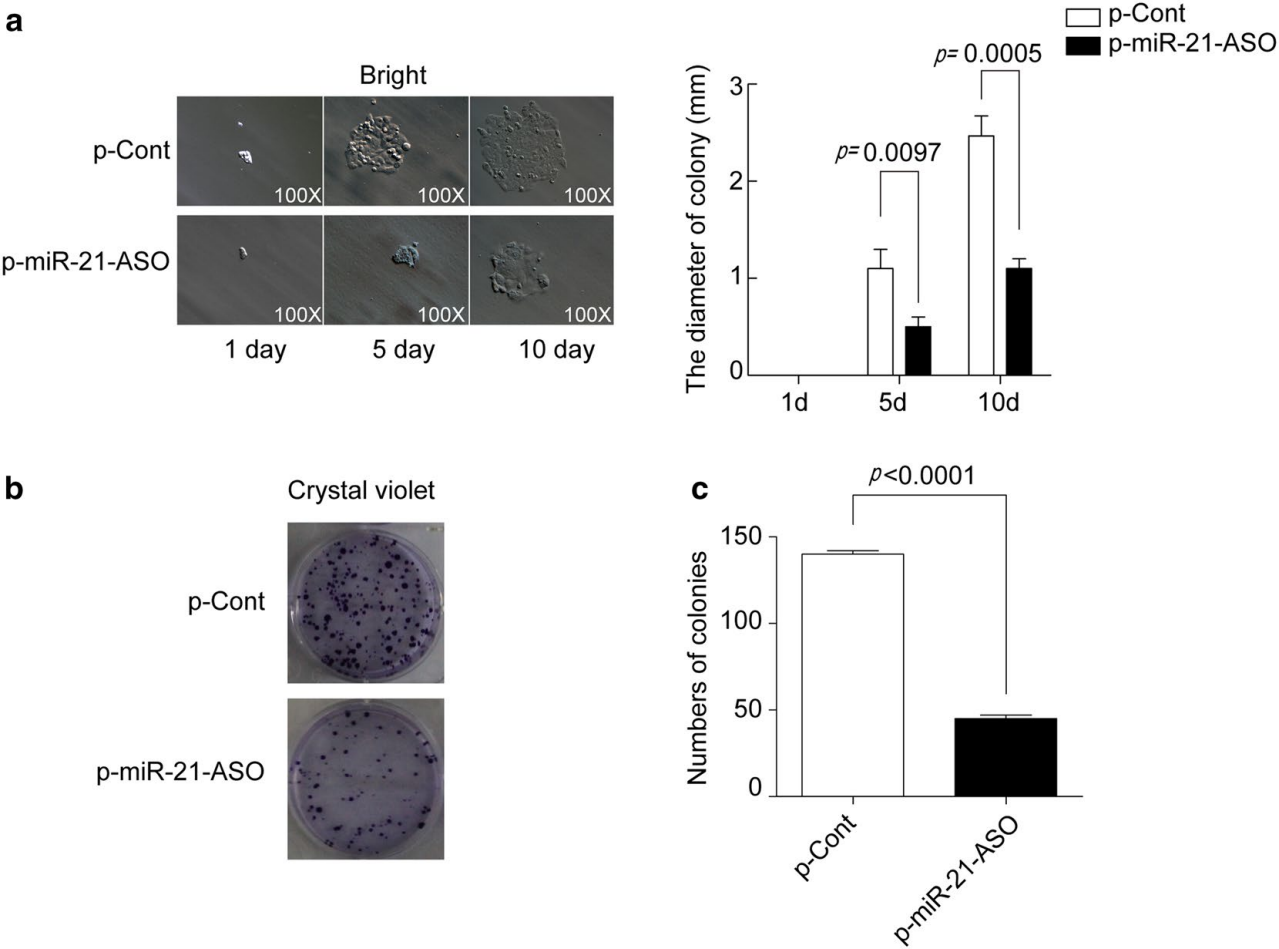
MiRNA-21 ASO reduced the colony formation capacity of human colon carcinoma cells. Human colon carcinoma cell line HCT116 cells were transiently transfected with p-miR-21-ASO or p-Cont (5 μg). **a** At indicated time point, the colony diameter was analyzed. **b** After 13 days, then colony numbers were analyzed by crystal staining and calculated (**c**). Data represent as mean ± SD of three independent experiments. *p < 0.05.

Next, we further investigate the possible effect of miR-21 ASO on the colony formation capacity of human colon carcinoma cells, which was closely related to the growth of cancer cells. As shown in Fig. 2, we found that both the volume and number of formative colonies of HCT116 cells in p-miR-21-ASO transfected group was also obviously decreased than those in p-Cont transfected group (p < 0.05), indicating that down-regulation of miR-21 by ASO also could impair the colony formation capacity of human colon carcinoma cells.

### MiRNA-21 ASO impaired the invasion and migration ability of human colon carcinoma cells

**Fig. 3:**
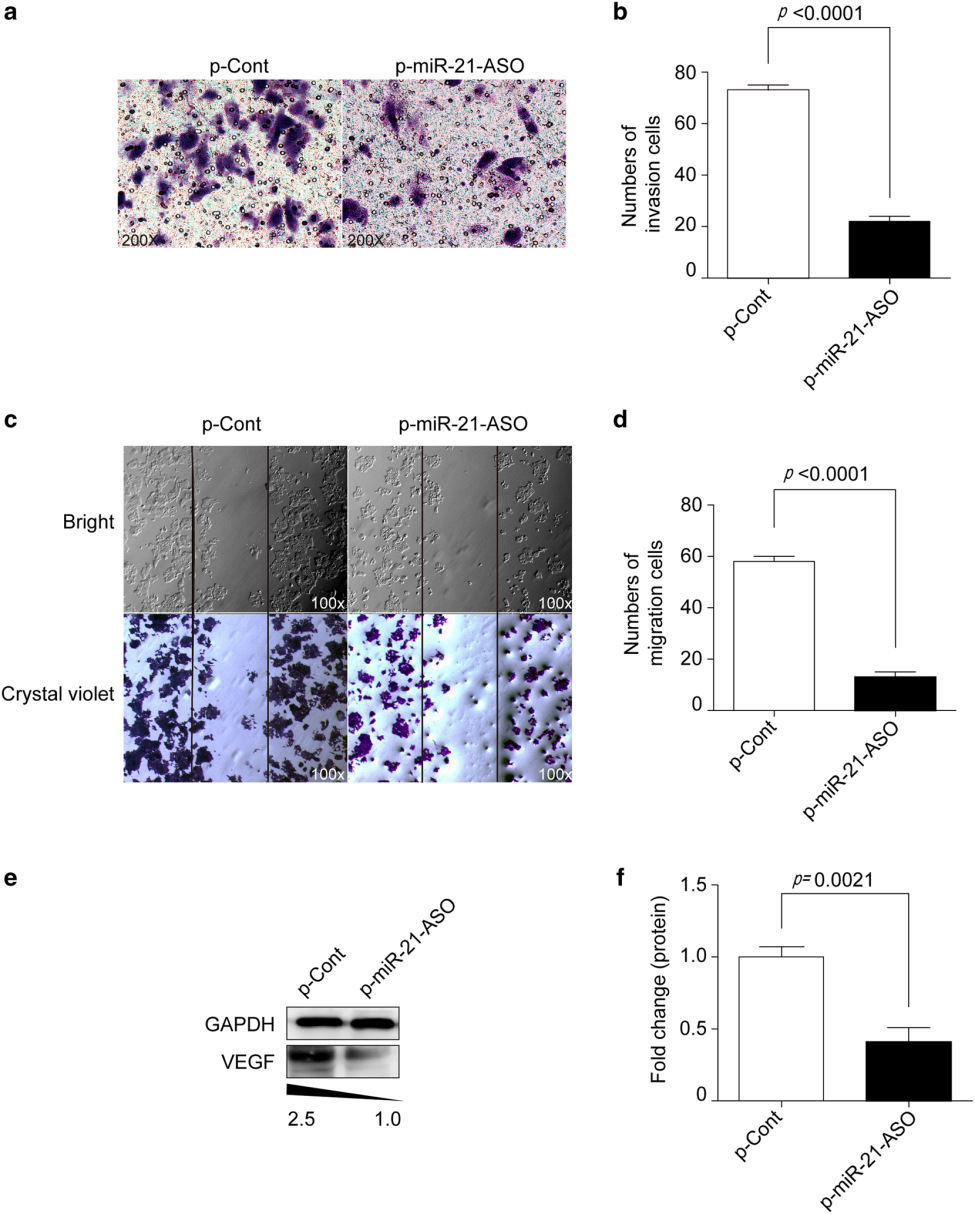
MiRNA-21 ASO impaired the invasion and migration ability of human colon carcinoma cells. Human colon carcinoma cell line HCT116 cells were transiently transfected with p-miR-21-ASO or p-Cont (5 μg). Then, the ability of invasion of cells was analyzed by Transwell assay (**a**) and calculated (**b**). **c** The ability of migration of cells also was determined by Wound-healing assay and calculated (**d**). **e** HCT116 cells were transiently transfected with p-miR-21-ASO or p-Cont (5 μg). 48 h later, the protein expression of VEGF was analyzed by western blotting and calculated (**f**). One representative of three experiments was shown. *p < 0.05.

Then, we further investigated whether down-regulation of miR-21 by miR-21 ASO could affect the invasion ability of colon cancer cells in vitro. As shown in Fig. 2a, b, transwell assay showed that the invasion cell number in p-miR-21-ASO transfected group was obviously decreased compared with that in p-Cont transfected group (Fig. 3a, b, p < 0.5). To analysis the possible effect of down-regulation of miR-21 on the migration capacity of colon carcinoma cells, scratch wound assay was also performed. Data showed that the migration capacity of HCT116 cells was also impaired in p-miR-21-ASO transfected group (Fig. 3c, d, p < 0.5).

Vascular endothelial growth factor (VEGF) is endothelial cell specific heparin-binding growth factor, which is critical for the proliferation and metastatic potential of colon carcinoma cells [13, 14]. We further detected the VEGF protein expression and found that its expression was decreased in p-miR-21-ASO transfected group compared with p-Cont transfected group (Fig. 3e, f, p < 0.5). Combining these data suggested that miR-21 ASO could impair the potential metastatic capacity of colon carcinoma cells.

### MiRNA-21 ASO reversed PTEN expression in human colon carcinoma cells

**Fig. 4:**
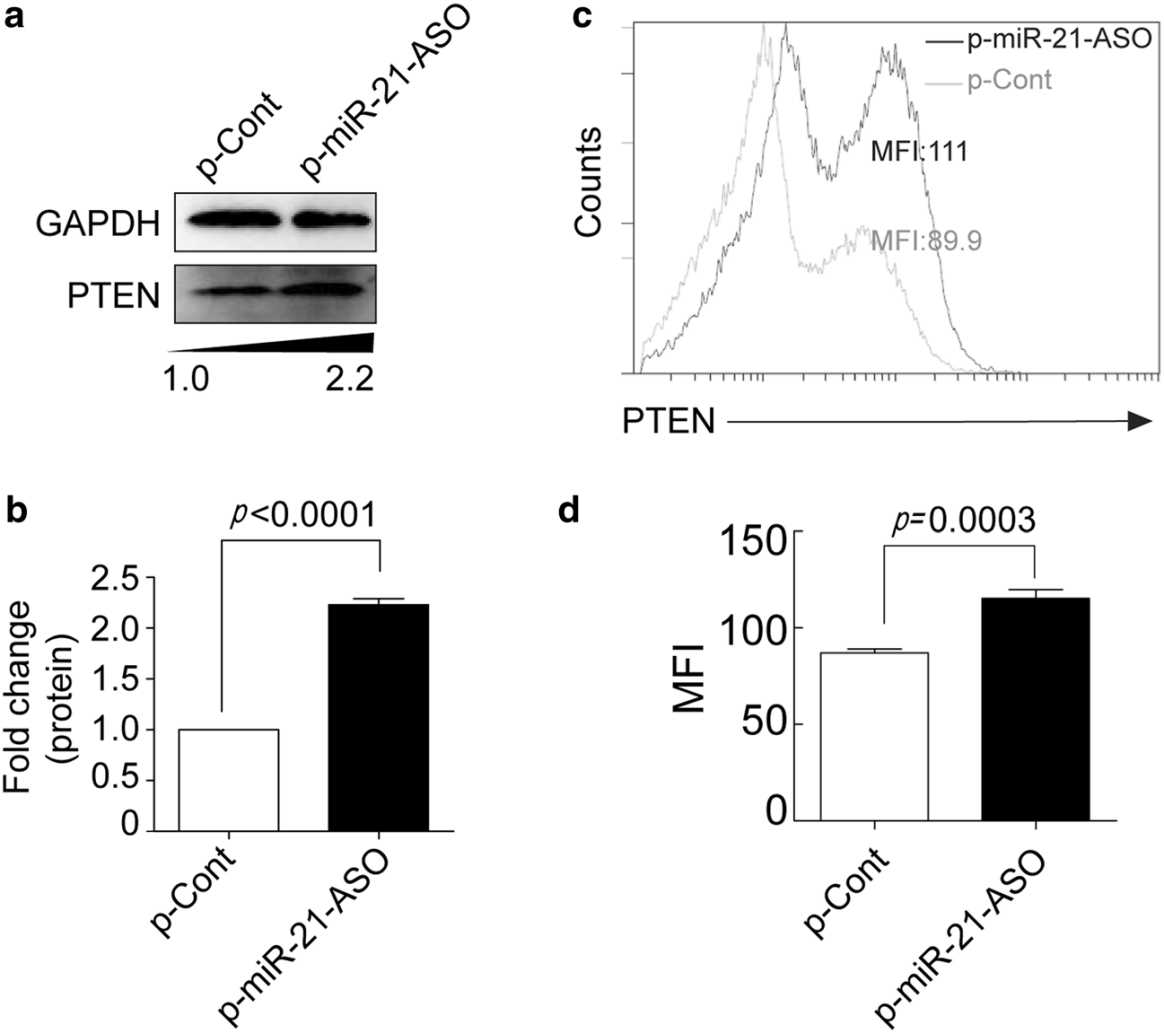
MiRNA-21 ASO reversed PTEN expression in human colon carcinoma cells. Human colon carcinoma cell line HCT116 cells were transiently transfected with p-miR-21-ASO or p-Cont (5 μg). 48 h later, the protein expression of PTEN was analyzed by Western blotting (**a**) and calculated (**b**). **c** The expression of PTEN also analyzed by FACS and then the mean fluorescence intensity (MFI) was calculated (**d**). *Gray line* p-Cont transfected group, *black line* p-miR-21-ASO transfected group. One representative of three experiments was shown. *p < 0.05.

Recent studies have shown that PTEN, a critical tumor suppressor in the occurrence and progression of various tumors, was one of target molecules of miR-21 [15–17]. Moreover, PTEN also could inhibit angiogenesis that associated with decreased expression of VEGF [18]. To investigate the possible mechanism of down-regulation of miRNA-21 by miR-21 ASO in the proliferation and migration of colon carcinoma, we further detected the expression of PTEN in human colon carcinoma HCT116 cells. As shown Fig. 4a, b, the expression of PTEN protein was significantly elevated in p-miR-21-ASO transfected group compared with that in p-Cont transfected group (*p* < 0.5). To further verify the expression of PTEN, we also analyzed the expression of PTEN protein in HCT116 cells using FACS analysis and similar result was obtained (Fig. 4c, d, *p* < 0.5).

### MiRNA-21 ASO altered the transduction of AKT and ERK pathways in human colon carcinoma cells

**Fig. 5:**
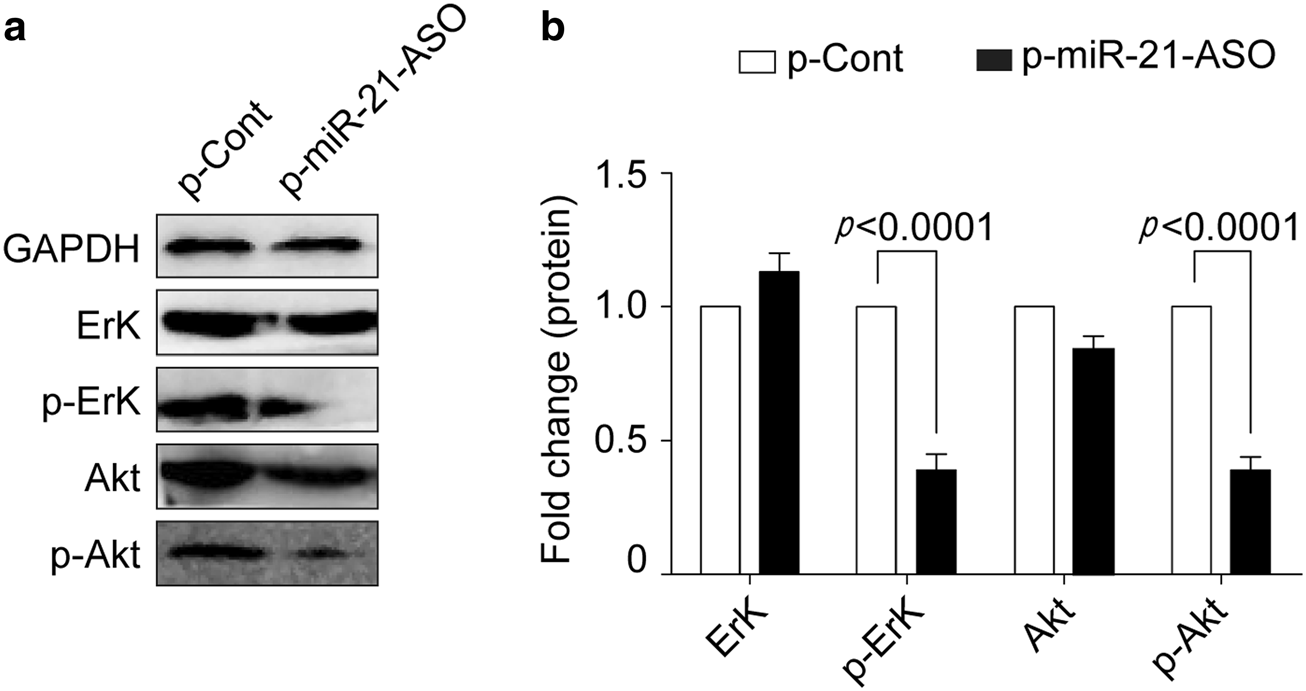
MiRNA-21 ASO altered the transduction of AKT and ERK pathways in human colon carcinoma cells. Human colon carcinoma cell line HCT116 cells were transiently transfected with p-miR-21-ASO or p-Cont (5 μg). 48 h later, the level of total and phosphor-AKT and phosphor-ERK1/2 were analyzed by Western blotting (**a**) and calculated (**b**). One representative of three experiments was shown.*p < 0.05.

Recent literatures demonstrated that transduction of AKT and ERK pathway was important for the growth and metastasis of various cancer cells including colon carcinoma cells [19, 20]. Moreover, PTEN also was closely related to the transduction of AKT and ERK pathway in various cancers [21]. Our above data showed that miR-21 ASO could elevate the expression of PTEN. Then, we further analyzed the possible change of transduction of AKT and ERK pathway in human colon carcinoma cells. Data showed that the level of total AKT and ERK did not show significant change in each group (Fig. 5a, b, p > 0.05). However, the level of phosphor-AKT in p-miR-21-ASO transfected group was decreased obviously (Fig. 5a, b, *p* < 0.05). Similarly, the level of phosphor-ERK1/2 was also decreased significantly (Fig. 5a, b, *p* < 0.05). These results suggested that down-regulated expression of miRNA-21 by miR-21 ASO could reverse the expression of PTEN and successively alter the transduction of AKT and ERK signaling pathways, which ultimately impaired the proliferation and metastasis potential of human colon carcinoma cells.

## Discussion

In present study, we firstly showed that miR-21 ASO could reduce the expression of miR-21 in human colon carcinoma cells. Moreover, miR-21 ASO also could impair the proliferation and colony formation capacity, as well as metastatic potential, of human colon carcinoma cells, which was related to altered expression of PTEN and successive transduction of AKT and ERK pathway. These data suggested that miR-21 might be a potential target for the therapeutic strategy against clinical colon carcinoma.

Accumulating literatures suggested that miR-21 was a critical regulator in the development of a various type of cancers [22–24]. Moreover, miR-21 was also reported as a potential biomarker for diagnosis and prognosis of clinical colon carcinoma [25, 26]. Recent researches further suggested that antisense oligonucleotides (ASO) against specific miRNA molecules might be a useful strategy for the development of biological therapy in clinical diseases including cancers. Such as, our recent evidence showed that ASO against miR-155, which was highly expressed in lung tissues in acute lung injury (ALI), could enhance the recovery of ALI [27]. To cancers, Li et al. reported that miR-150 ASO could inhibit proliferation of lung cancer cells by regulating miR-150 expression [28]. Moreover, Qiang et al. demonstrated that ASO against miR-20a could inhibit the invasion and migration of prostate cancer cells [29]. In present study, we found that miR-21 ASO could reduce the expression of miR-21 in human colon carcinoma cells. Moreover, the growth and colony formation capacity of colon carcinoma cells also were significantly impaired. Similarly, Li et al. reported that miR-21 ASO could abrogate the expression of miR-21 and reduce the growth of EGFR-TKI-sensitive human lung adenocarcinoma cells [30]. These data suggested that miR-21 might be a useful target for the development of therapeutic strategy against colon carcinoma. Therefore, successive research work on the effect of miR-21 ASO in the growth of human colon carcinoma cells in vivo was much valuable for the validation of the development of miR-21 targeted based therapeutic strategy against colon carcinoma.

PTEN is a discovered well-known tumor suppressor gene and involved in the regulation of various type of cancers including colon carcinoma [31–33]. For instance, Setia et al. reported that the expression of PTEN was significantly decreased in carcinogenic condition in colon cancer [34]. Jaqan et al. further showed that overexpression of PTEN could abrogate the dissemination and growth of colon carcinoma cells [35]. In this study, we found that miR-21 ASO could reverse the expression of PTEN, which was a target of miR-21, accompanied by reduced metastatic potential of colon carcinoma cells. Furthermore, the transduction of AKT and ERK pathway also were altered. Consistently, Setia et al. found that the transduction of AKT and ERK pathway was elevated in colon cancer [36]. Auyeung et al. further showed that inhibition of AKT and ERK pathway transduction could induce the apoptosis of colon cancer cells [37, 38]. Most recently, Sun et al. reported that PTEN could reduce the proliferation of colon carcinoma through regulating the transduction of AKT pathway [39]. In addition, some studies reported that PTEN also could regulate the expression of VEGF, which was important for the carcinogenesis of cancers [40, 41]. Such as, Tian et al. reported that PTEN could regulate the expression of VEGF through AKT pathway in human hepatoblastoma cells [18]. Similarly, we also found that miR-21 ASO could reduce the expression of VEGF in colon carcinoma cells. Therefore, combing these data further highlighted the critical role of PTEN pathway in the development of colon carcinoma. Taken together, we presumed that miR-21 ASO could reverse the expression of PTEN and successively alter the transduction of AKT and ERK pathway, accompanied by reduced expression of VEGF. Finally, it should be pointed out that we did not exclude the potential contribution of other target molecules of miR-21, which did not been investigated in present study, to the effect of miR-21 ASO on the proliferation and migration capacity of colon carcinoma cells. In fact, successive research work on these target molecules including PDCD4 [42], was also important for the elucidation of effect of miR-21 ASO on colon carcinoma cells.

In summary, our study showed miR-21 ASO could effectively reduce the expression of miR-21 and successively impair the proliferation and migration of human colon carcinoma cells, which was closely related to altered expression of PTEN and transduction of AKT and ERK pathway, indicating that miR-21 might be a potential target and be useful for the development of new therapeutic strategy against clinical colon carcinoma.

## Methods

### Materials

McCoy 5A was purchased from Sigma. T4 DNA ligase was purchased from Fermentas. The pcDNA™6.2-GM/EmGFP-miR, pcDNA™6.2-GM/EmGFP-miR-neg-control plasmid and Lipofectamine 2000 were purchased from Invitrogen. Trizol reagent was purchased from Takara. RevertAid First Strand cDNA Synthesis kits were purchased from Thermo. Antibodies against VEGF, PTEN, AKT, and phospho-AKT were purchased from Abcam. Antibodies against GAPDH, ERK1/2 and phospho-ERK1/2 were purchased from Cell Signaling Technology. Cell counting kit-8 reagent was purchased from Boster. Transwell chambers were purchased from Costar. SYBR^®^ Premix Ex Taq™ II was purchased from Takara. C1000™Thermal Cycler and S1000™Thermal Cycler were purchased from BIO-RAD. Flow cytometry from Beckman Coulter.

### Vector construction

Designed antisense oligonucleotides targeting mature miRNA-21 sequence (UAGCUUAUCAGACUGAUGUUGA), sense strand: 5′-TGCTTCAACATCAGTCTGATAAGCTATTTTTG-3′, antisense strand: 5′-CCTGCAAAAATAGCTTATCAGACTGATGTTGA-3′. The pcDNA-6.2-miRNA-21-ASO vector was constructed through annealing synthesized ds oligonucleotides connected to pcDNA™6.2-GM/EmGFP-miR. Plasmid sequences were confirmed by sequencing.

### Cell culture and transient transfection

Human colon carcinoma cell lines HCT-116, HT29, SW620 and normal colonic cell line FHC were obtained from National Rodent Laboratory Animal Resource (Shanghai, China). All the cancer cells were cultured in McCoy 5A, RPMI-1640 or Leibovitz’s L-15 medium containing 100 IU/mL penicillin, 100 μg/mL streptomycin, 20 mM glutamine and 10% heat-inactivated fetal bovine serum (FBS). All cells were cultured in a humidified atmosphere of 5% CO_2_ at 37°C. For transfection, cells were seeded at 70% confluence and 12 h later, cells were transfected with pcDNA-6.2-miRNA-21-ASO vector or pcDNA6.2-miR-neg-control vector with Lipofectamine 2000 according to the manufacturer’s instruction. Cells were harvested after indicated time for following experiments.

### Quantitative Real-time PCR for miRNA-21

Total RNA was extracted from cells with Trizol and reverse transcribed using RevertAid First Strand cDNA Synthesis kits according to the manufacturer’s instructions. The resulting complementary DNA (cDNA) was used for real-time PCR using the SYBR^®^ Premix Ex Taq™ II with triplicates. Data collection was performed on the CFX96™ Real-Time System. All calculations were normalized to an endogenous control, GAPDH. The relative quantitation value for the target gene compared to its calibrator is expressed as 2^−ΔΔCt^. Aliquots of reaction mixture following conditions: initial denaturation at 95°C for 5 min followed by 40 cycles of 95°C for 15 s, 60°C for 30 s.

### Cell counting kit-8 assay

HCT116 cells/SW620 cells were seeded in 96-well plates at 1 × 10^4^/well with triplicate and infected with pcDNA-6.2-miRNA-21-ASO (p-miR-21-ASO) or pcDNA6.2-miR-Ctrl (p-Cont). At indicated time points, cells were detected using cell counting kit-8 (CCK-8) assay. 20 µL CCK-8 solution was added into each well. After 3 h of incubation at 37°C. The absorbance was measured with a spectrophotometer at 450 nm with 600 nm as a reference.

### Colony formation assay

Collected infected 72 h HCT116 cells as above described. Cells were trypsinized to single cell suspension and seeded in 6-well plates at 1,000/well for clone forming experiment. Then, the cells were incubated in a humidified atmosphere of 5% CO_2_ at 37°C. The medium were renewed every 5 days. 13 days later, the colonies were stained with crystal violet and the colony diameter and number was statistically analyzed.

### Cell invasion and migration assay

Cell invasion was performed by Matrigel invasion assay. 8-mm pore size-culture inserts were first coated with Matrigel (BD Bioscience). HCT116 cells transfected with p-miR-21-ASO or p-Cont for 48 h were harvested, suspended (5 × 10^4^/well) in 200 µL serum-free medium and then seeded on the upper compartment of chamber. The lower chamber was added 500 µL McCoy 5A media with 10% FBS. After 48 h incubation, the cells in the bottom chamber that had invaded were stained with crystal violet and counted using fluorescence microscopy (100× magnification). In addition, Wound healing assay was also performed for analysis of cell migration in vitro. Cells were cultured as previously described. Then, a single scratch was made in the center of cell monolayer using a 1,000 tip. The scratch areas were visualized under fluorescence microscope with a magnification 100× and the migrated cells were counted. Three independent experiments were performed with triplicate wells.

### Western blotting

Cells were lysed with RIPA lysis buffer [1 mM phenylmethylsulfonyl fluoride (PMSF), 1× Protease Inhibitors, 1× Phosphatase Inhibitors] on ice for 30 min. Total cellular proteins were assayed using Bio-Rad protein assay reagent. Equal amounts of protein were subjected to SDS-PAGE electrophoresis, then electrophoretic transfer to nitrocellulose membranes. Membranes were blocked with 5% nonfat dry milk in PBS with 0.1% Tween20 for 1 h at 37°C, Then incubated with antibodies VEGF, PTEN, phosphor-AKT, total AKT, phospho-ERK1/2, and total ERK1/2 at 4°C for overnight. Finally, incubated with horseradish peroxidase-conjugated secondary antibodies for 1 h. Results were analyzed by ECL detection system.

### FACS analysis on PTEN expression

Collected infected 72 h HCT116 cells and then fixed with 4% paraformaldehyde for 10 min and then flushing with twice. Cells were blocked with 5% nonfat dry milk in PBS with 0.1% Tween 20, and incubated with antibodies against PTEN for 30 min at 22°C after flushing with twice. The secondary antibody used was Alexa Fluor^®^ 488 goat anti-rabbit IgG (H + L) at 1/500 dilution for 30 min at 22°C. Eventually the stained cells were analyzed by a flow cytometer.

### Statistical analysis

All values were represented as the mean ± SD from at least three independent experiments. Student’s T-test for two groups or one-way analysis of variance (ANOVA) for three or more groups were performed to evaluate the statistical significance by using GraphPad Prism 5 software. P values less than 0.05 were considered statistically significant.

## Additional files

Additional file 1:
**Figure S1.** The relative expression of miR-21 in colon carcinoma cells. Three human colon carcinoma cells and normal colonic cells FHC were culture in 96-well plate. Then, the relative expression of miR-21 was determined by real-time PCR assay. *p < 0.05. Additional file 2:
**Figure S2.** The effect of miR-21 ASO on the growth of human colon carcinoma cell line SW620 cells in vitro. Human colon carcinoma cell line SW620 cells were transiently transfected with p-miR-21-ASO or p-Cont (5 μg). (A) 48 h later, the relative expression of miR-21 was analyzed by real-time PCR assay. (B) The growth of cells was determined by CCK-8 assay at indicated tie point. One representative of three experiments is shown. *p < 0.05.
