# Supplementary material for: Antisense oligonucleotides against microRNA-21 reduced the proliferation and migration of human colon carcinoma cells
Source: Cancer Cell Int. 2015 Aug 1;15:77. doi: 10.1186/s12935-015-0228-7 (PMC4522075; doi:10.1186/s12935-015-0228-7)
Supplement: Additional file 1: — Figure S1. The relative expression of miR-21 in colon carcinoma cells. Three human colon carcinoma cells and normal colonic cells FHC were culture in 96-well plate. Then, the relative expression of miR-21 was determined by real-time PCR assay. *p < 0.05. [file 12935_2015_228_MOESM1_ESM.pdf]

Supplementary data figure 1

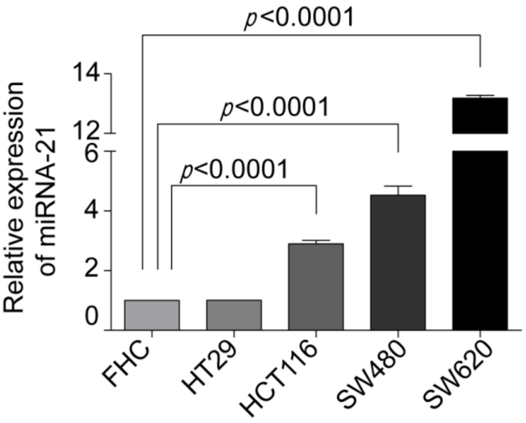

Supplementary data figure 1. The relative expression of miR-21 in colon carcinoma cells. Three human colon carcinoma cells and normal colonic cells FHC were culture in 96-well plate. Then, the relative expression of miR-21 was determined by real-time PCR assay. *p<0.05.
