# Supplementary material for: Antisense oligonucleotides against microRNA-21 reduced the proliferation and migration of human colon carcinoma cells
Source: Cancer Cell Int. 2015 Aug 1;15:77. doi: 10.1186/s12935-015-0228-7 (PMC4522075; doi:10.1186/s12935-015-0228-7)
Supplement: Additional file 2: — Figure S2. The effect of miR-21 ASO on the growth of human colon carcinoma cell line SW620 cells in vitro. Human colon carcinoma cell line SW620 cells were transiently transfected with p-miR-21-ASO or p-Cont (5 μg). (A) 48 h later, the relative expression of miR-21 was analyzed by real-time PCR assay. (B) The growth of cells was determined by CCK-8 assay at indicated tie point. One representative of three experiments is shown. *p < 0.05. [file 12935_2015_228_MOESM2_ESM.pdf]

Supplementary data figure 2

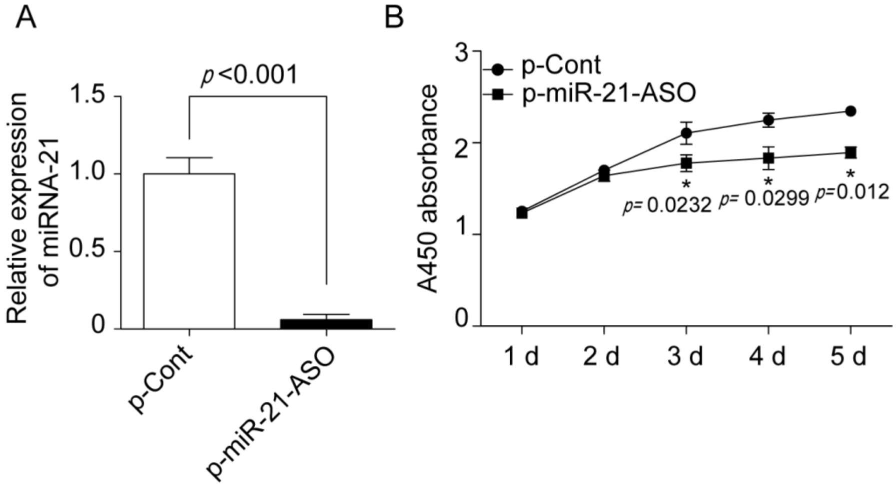

Supplementary data figure 2. The effect of miR-21 ASO on the growth of human colon carcinoma cell line SW620 cells in vitro. Human colon carcinoma cell line SW620 cells were transiently transfected with p-miR-21-ASO or p-Cont (5ug). (A) 48h later, the relative expression of miR-21 was analyzed by real-time PCR assay. (B) The growth of cells was determined by CCK-8 assay at indicated tie point. One representative of three experiments is shown. *p<0.05.
